# Supplementary material for: Population replacement gene drive characteristics for malaria elimination in a range of seasonal transmission settings: a modelling study
Source: Malar J. 2022 Jul 26;21:226. doi: 10.1186/s12936-022-04242-2 (PMC9327287; doi:10.1186/s12936-022-04242-2)
Supplement: Supplementary file 1 — Additional file 1: Figure S1. Distribution of vector migration distances within the simulated area. Fraction of vector migrations versus distance, computed by summing total migration distance over each migrating vector’s existence within a 2-month period (August 1 to October 1 in the first simulation year with annual EIR = 30 and no ITNs or gene drive release), counting the number of total migration distances within each histogram distance bin, and then dividing by the total number of migrating vectors in the 2-month period. Total migration distance as plotted here does not necessarily represent the distance between a vector’s starting and ending point (i.e., its displacement), but instead represents the total distance traveled. Migration probabilities are governed by an empirical negative exponential distance decay function [48]. Figure S2. Screenshots of accompanying website for interactive visualization of simulation output. Screenshots of two different tabs on the website located here: https://gene-drive.bmgf.io. Website users can interactively visualize the effects of tested gene drive parameters on elimination probabilities, elimination timing, prevalence, vector populations, and allele frequencies over all simulated combinations of gene drive release types, ITN deployments, and transmission regimes. Figure S3. Representative time series illustrating how elimination probabilities can either increase or decrease with increasing fitness costs of complete construct expression. Time series of malaria prevalence, total adult vector population, infectious vector fraction, total infectious adult vector population, and adult vector effector frequency over increasing values of fitness costs associated with complete construct expression (sne). Elimination probabilities (e.p.) and number of days to elimination (e.d.) are denoted in the subplot titles. In the simulations corresponding to these time series, classic gene drive mosquitoes were released in a moderate transmis [file 12936_2022_4242_MOESM1_ESM.pdf]

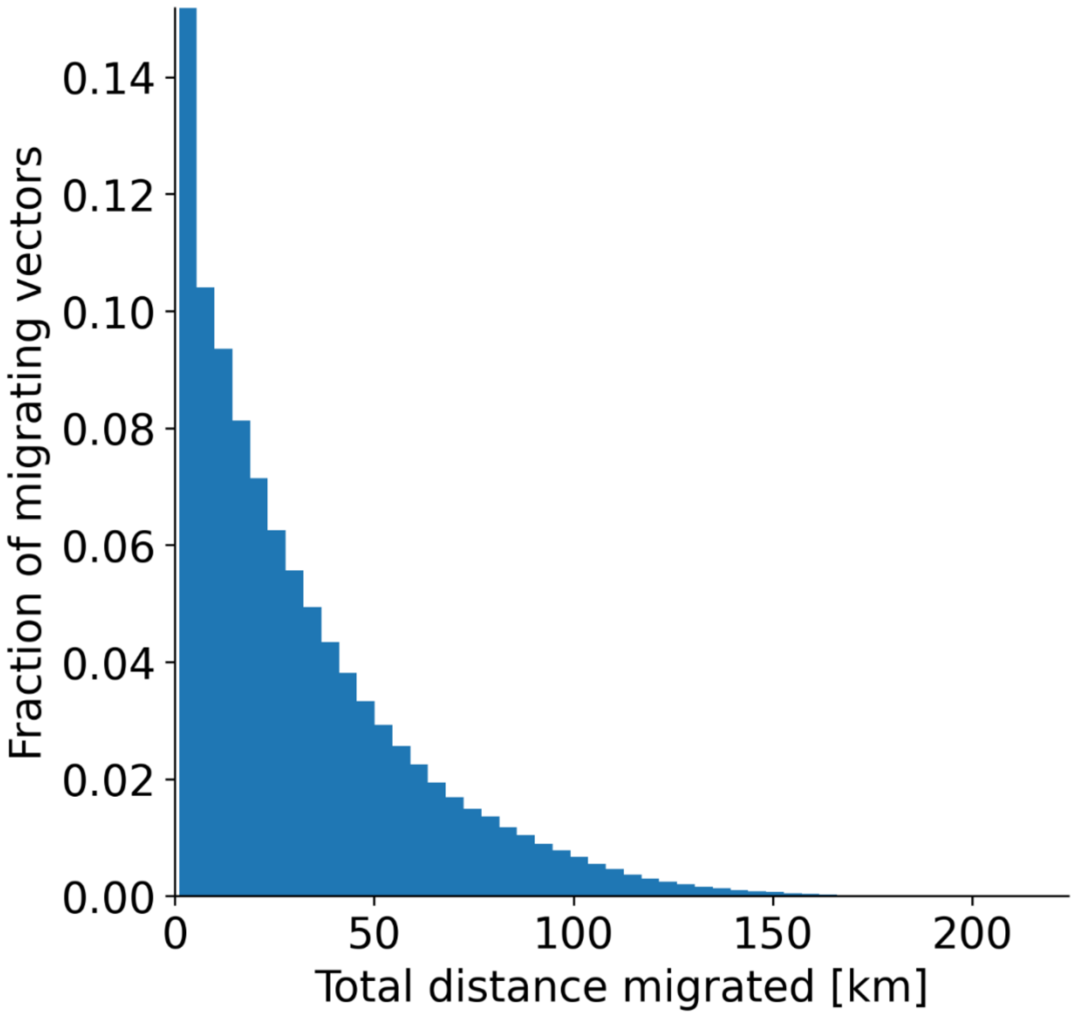

Figure S1 Distribution of vector migration distances within the simulated area.

Fraction of vector migrations versus distance, computed by summing total migration distance over each migrating vector's existence within a 2-month period (August 1 to October 1 in the first simulation year with annual EIR = 30 and no ITNs or gene drive release), counting the number of total migration distances within each histogram distance bin, and then dividing by the total number of migrating vectors in the 2-month period. Total migration distance as plotted here does not necessarily represent the distance between a vector's starting and ending point (i.e, its displacement), but instead represents the total distance traveled. Migration probabilities are governed by an empirical negative exponential distance decay function [48].

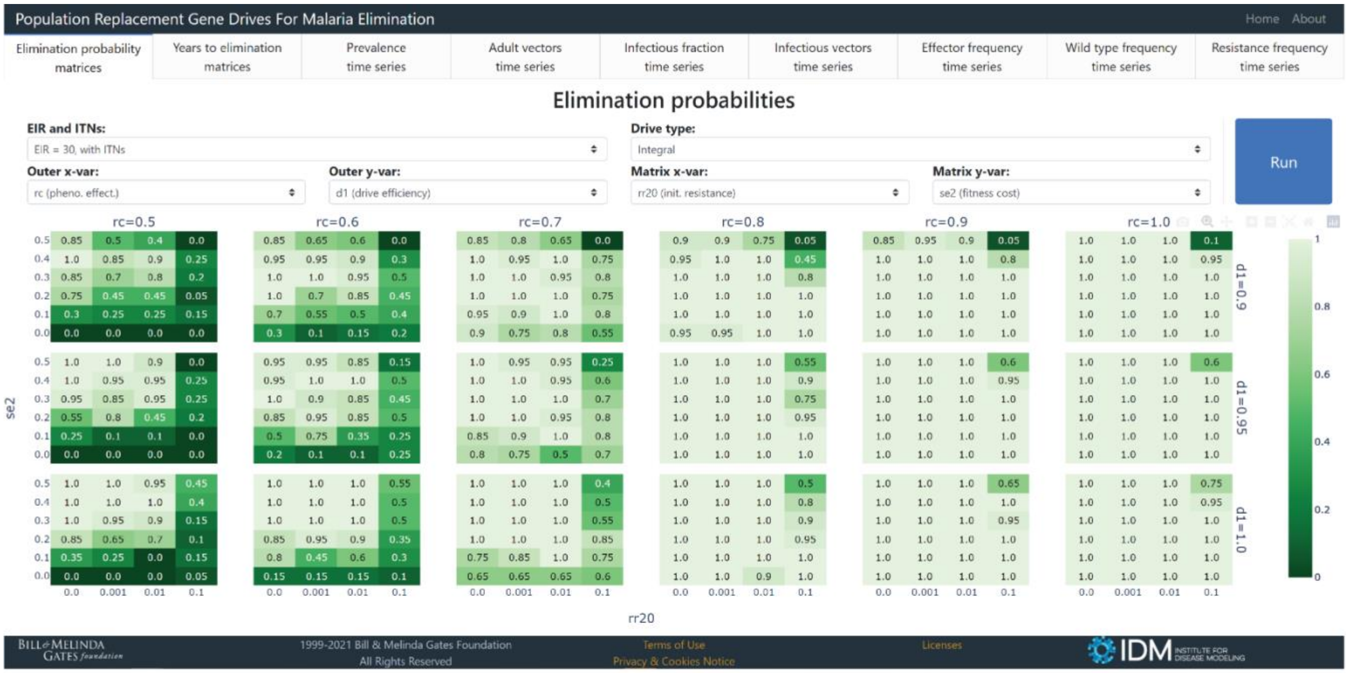

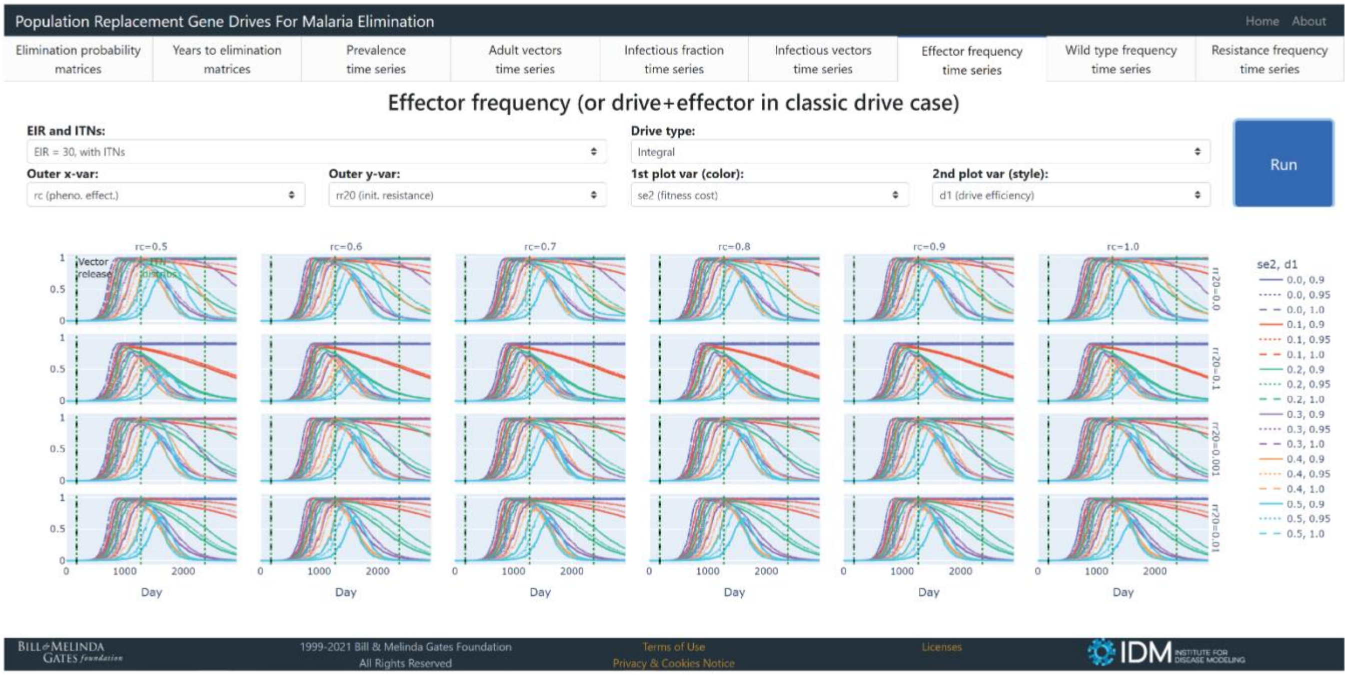

Figure S2 Screenshots of accompanying website for interactive visualization of simulation output.

Screenshots of two different tabs on the website located here: https://gene-drive.bmgf.io. Website users can interactively visualize the effects of tested gene drive parameters on elimination probabilities, elimination timing, prevalence, vector populations, and allele frequencies over all simulated combinations of gene drive release types, ITN deployments, and transmission regimes.

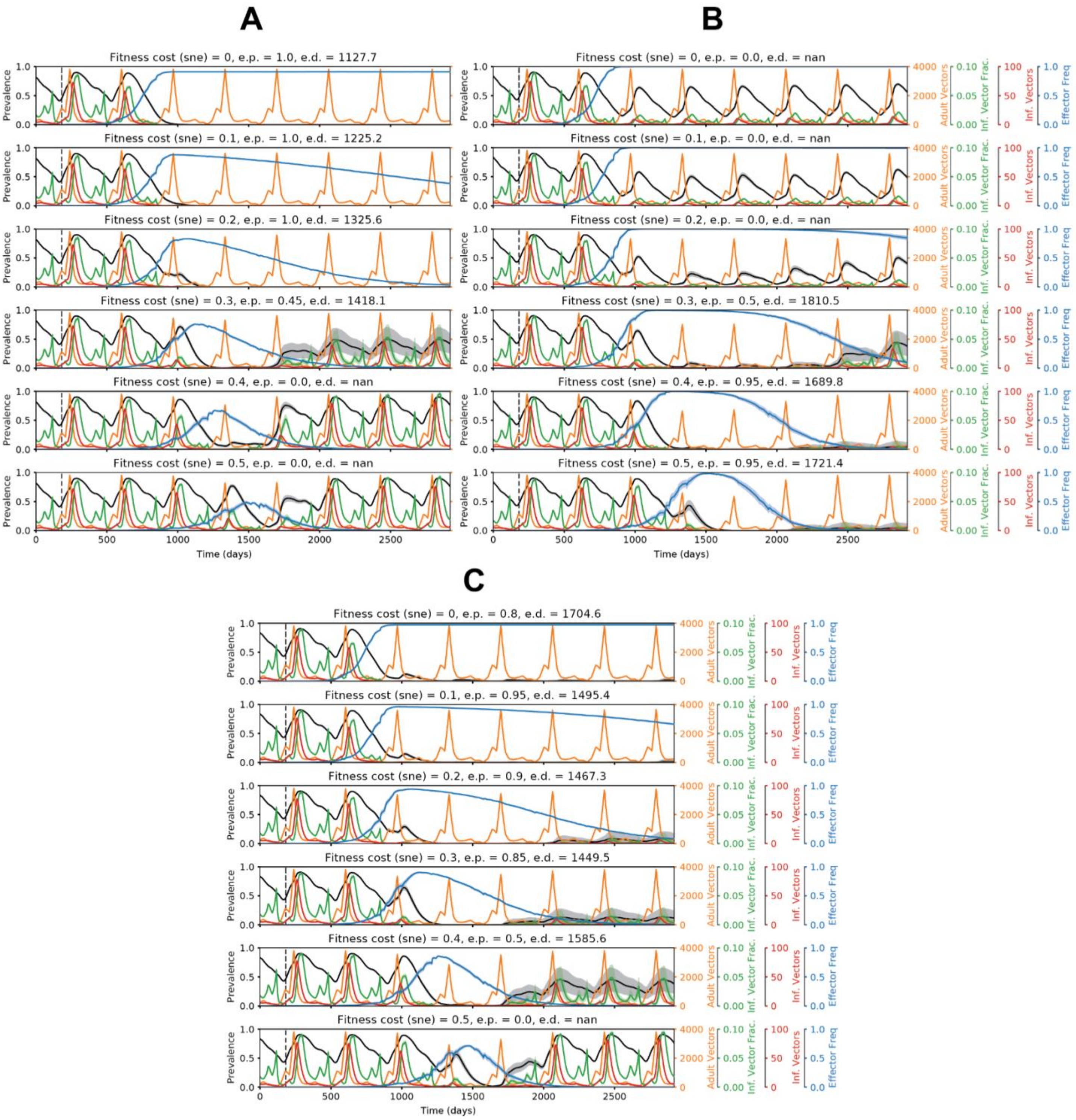

Figure S3 Representative time series illustrating how elimination probabilities can either increase or decrease with increasing fitness costs of complete construct expression.

Time series of malaria prevalence, total adult vector population, infectious vector fraction, total infectious adult vector population, and adult vector effector frequency over increasing values of fitness costs associated with complete construct expression (*sne*). Elimination probabilities (e.p.) and number of days to elimination (e.d.) are denoted in the subplot titles. In the simulations corresponding to these time series, classic gene drive mosquitoes were released in a moderate transmission setting (annual EIR = 30). In column A, representing the case in which increasing fitness costs increase elimination probabilities, non-*sne* parameters were set equal to the following values: drive efficiency (*d*) = 1, pre-existing resistance (*rr0*) = 0.001, and transmission-blocking effectiveness (*rc*) = 0.7. In column B, representing the case in which increasing fitness costs decrease elimination probabilities, non-*sne* parameters were set equal to the following values: *d* = 1, *rr0* = 0.1, and *rc* = 1. In column C, representing the case in which increasing fitness costs increase and then decrease elimination probabilities, non-*sne* parameters were set equal to the following values: *d* = 0.95, *rr0* = 0.01, and *rc* = 0.9. In column A, the higher the fitness costs, the lower the total vector population, and the greater the chance of locally eliminating malaria. In column B, the higher the fitness costs, the lower the peak effector frequency, and the lower the chance of elimination. In column C, the effects described for columns A and B are both at play.

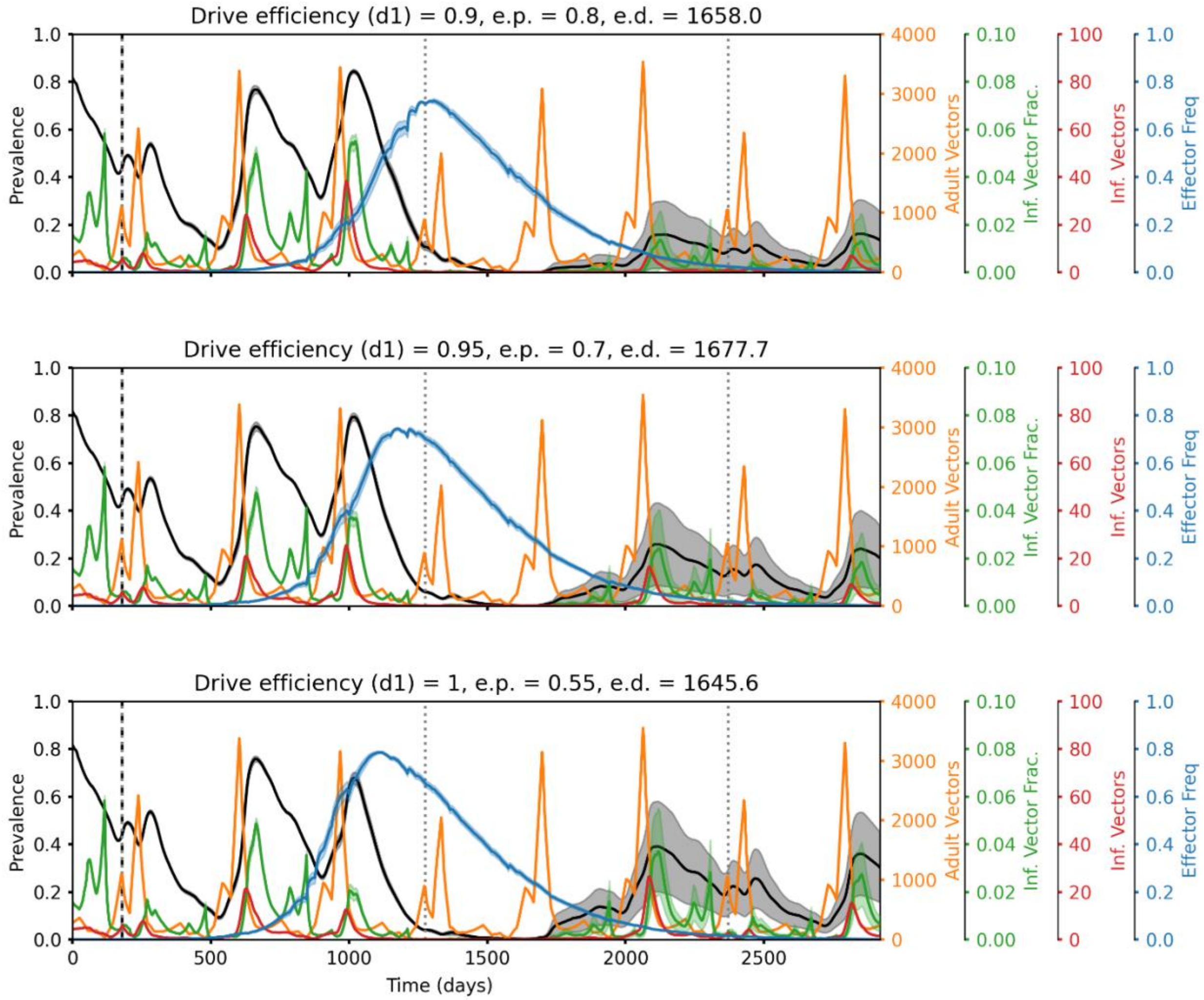

Figure S4 Representative time series illustrating how elimination probabilities can sometimes decrease with increasing drive efficiency.

Time series of malaria prevalence, total adult vector population, infectious vector fraction, total infectious adult vector population, and adult vector effector frequency over increasing values of drive efficiency (*d1*). Elimination probabilities (e.p.) and number of days to elimination (e.d.) are denoted in the subplot titles. In the simulations corresponding to these time series, integral gene drive mosquitoes were released and ITNs were deployed in a moderate transmission setting (annual EIR = 30) with non-*d1* parameters set equal to the following values: transmission-blocking effectiveness (*rc*) = 0.7, pre-existing resistance at the effector target site (*rr20*) = 0.1, and fitness cost of expressing the effector (*se2*) = 0.3. The higher the drive efficiency, the earlier the peak in effector frequency, and the lower the chance of locally eliminating malaria when this earlier peak does not match up with maximum ITN efficacy.

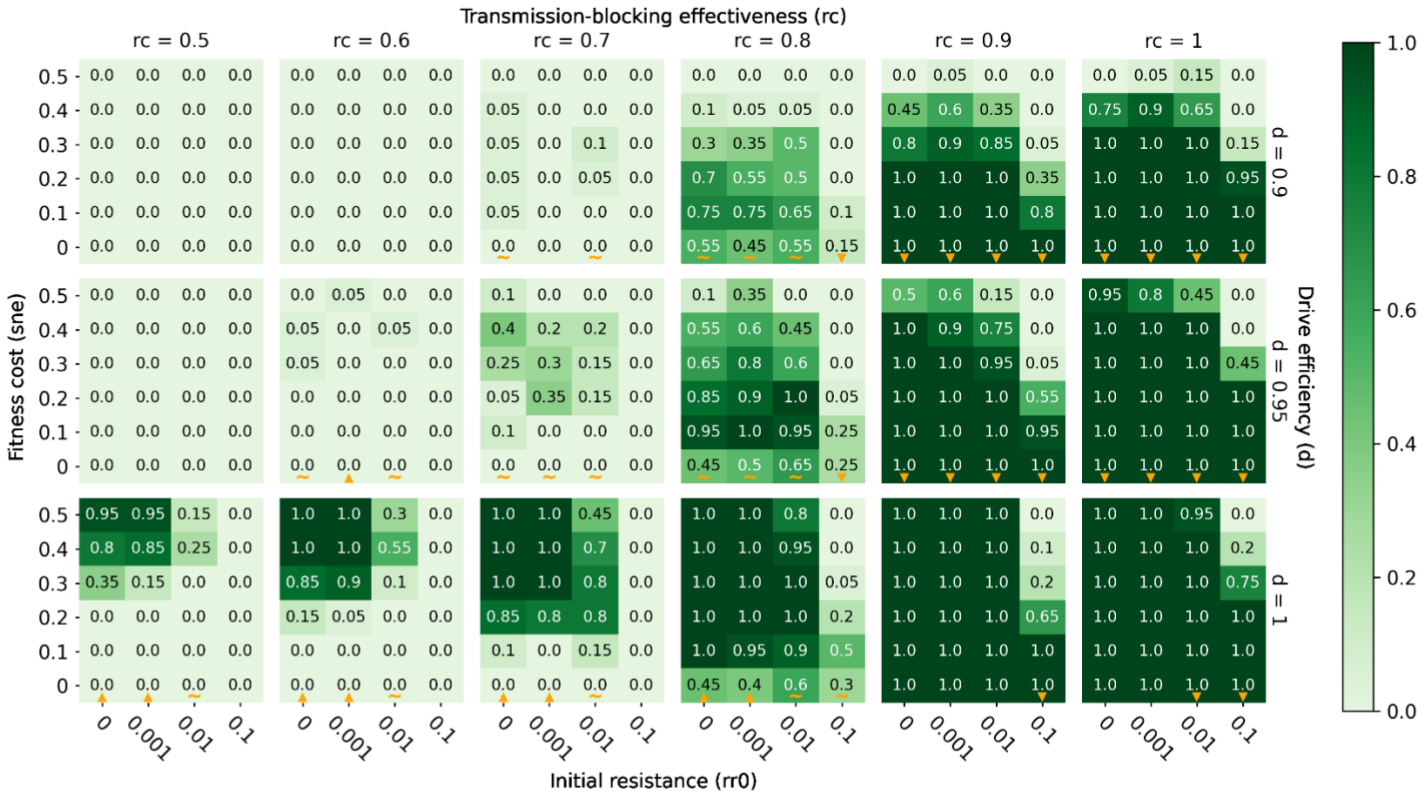

Figure S5 Elimination probabilities after a single release of classic gene drive mosquitoes only in a low transmission (annual EIR = 10) regime.

Same as Figure 3.

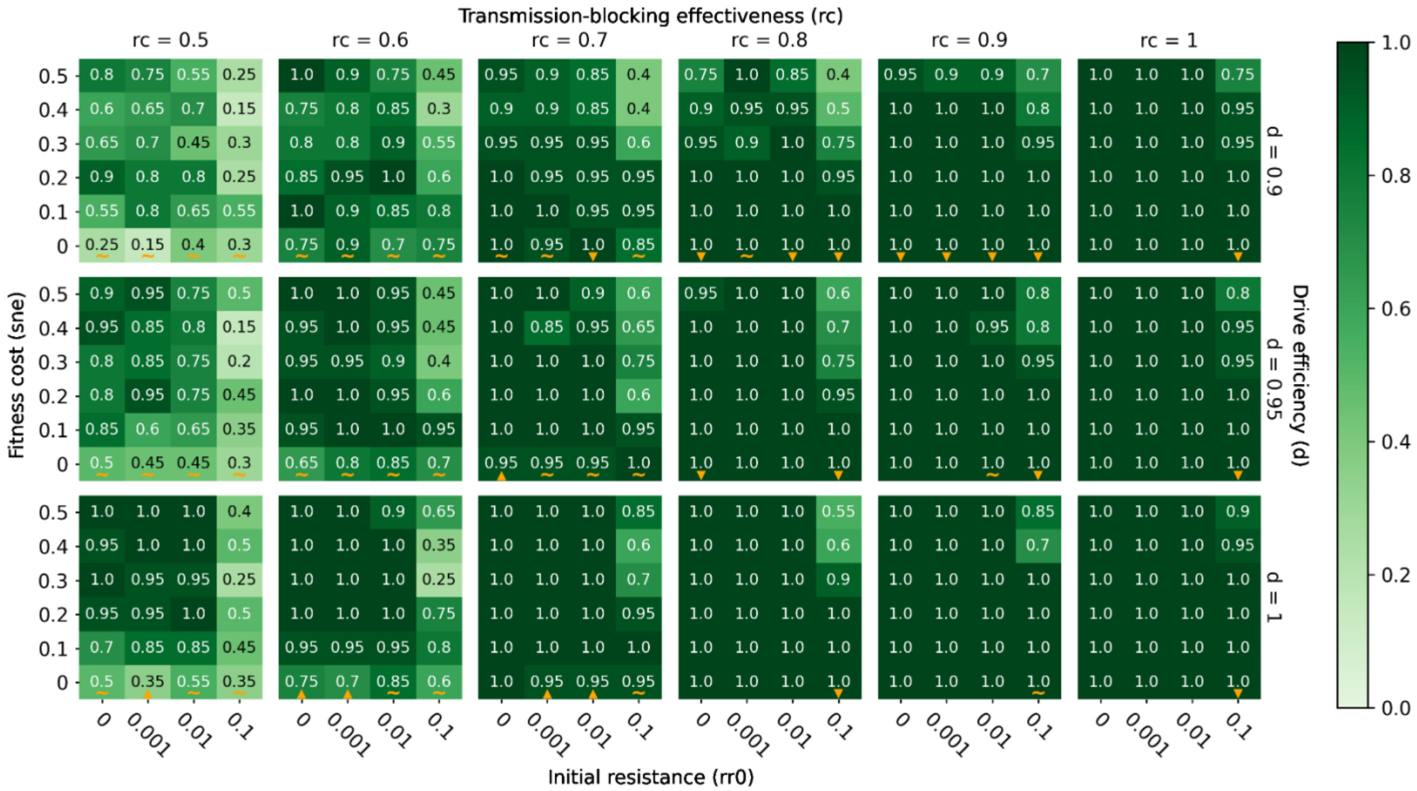

Figure S6 Elimination probabilities after a single release of classic gene drive mosquitoes and ITN deployment in a low transmission (annual EIR = 10) regime.

Same as Figure 3.

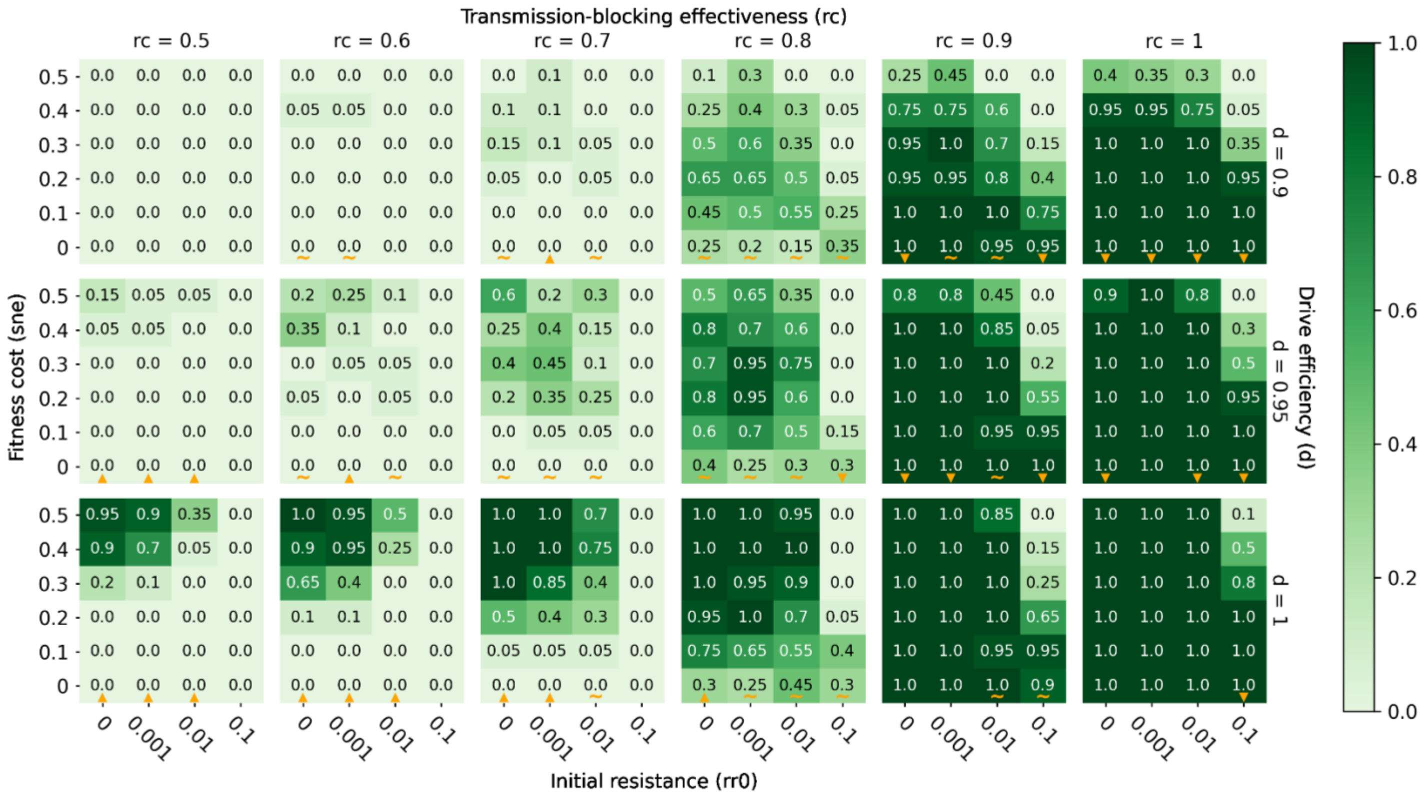

Figure S7 Elimination probabilities after a single release of classic gene drive mosquitoes and ITN deployment in a high transmission (annual EIR = 80) regime.

Same as Figure 3.

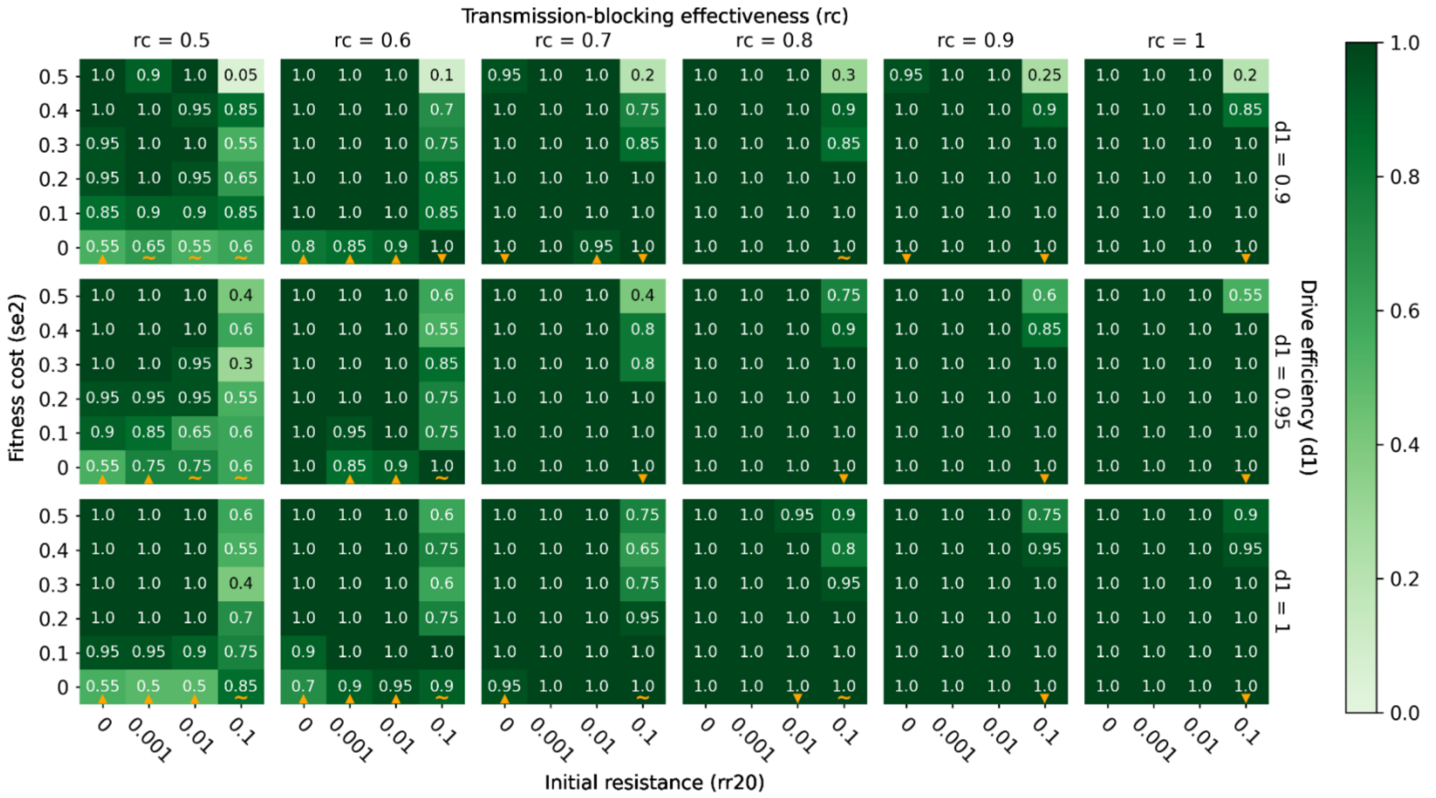

Figure S8 Elimination probabilities after a single release of integral gene drive mosquitoes and ITN deployment in a low transmission (annual EIR = 10) regime.

Same as Figure 3.

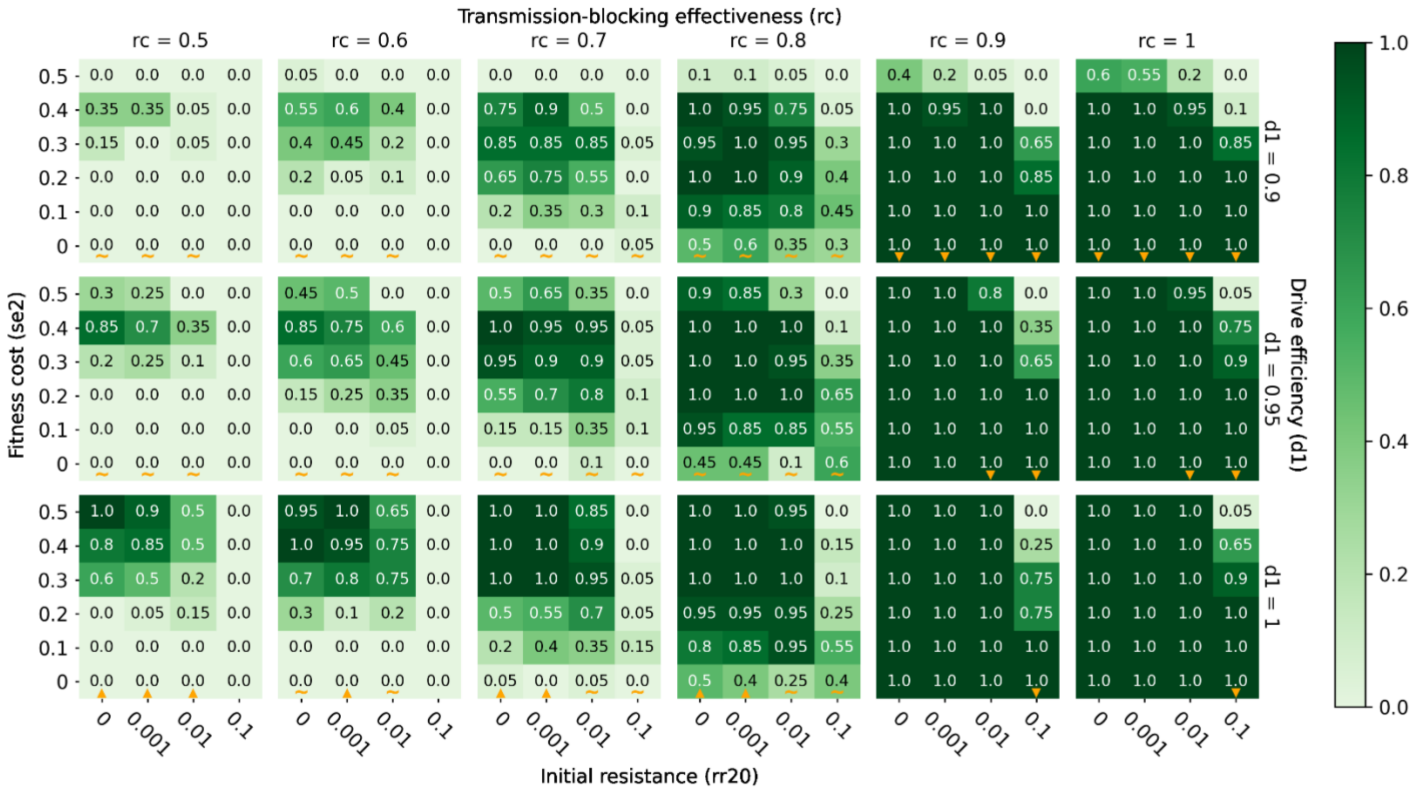

Figure S9 Elimination probabilities after a single release of integral gene drive mosquitoes and ITN deployment in a high transmission (annual EIR = 80) regime.

Same as Figure 3.

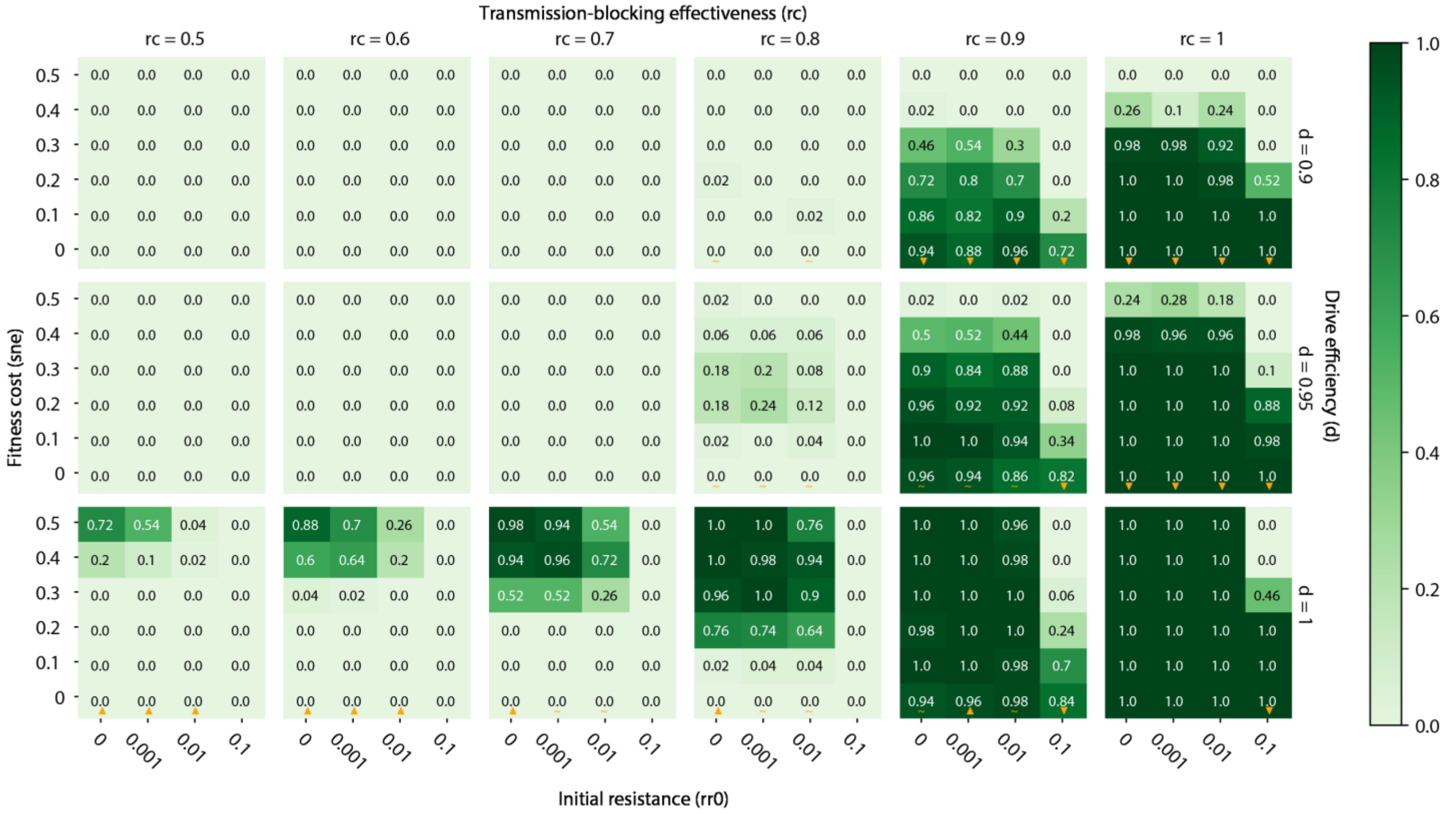

Figure S10 Elimination probabilities after a single release of classic gene drive mosquitoes only in a low transmission (annual EIR = 30) regime. We run 50 seeds per parameter set.

Same as Figure 3.
